# Cultural Cuisines and Cardiovascular Care: Tailoring Dietary Interventions for Effective Prevention

**DOI:** 10.1016/j.advnut.2024.100331

**Published:** 2024-10-29

**Authors:** Alina Yang

**Affiliations:** Scarsdale High School, USA

Dear Editor:

I read with great interest the review article entitled “Dietary Patterns and Cardiovascular Diseases in Asia: A Systematic Review and Meta-Analysis” [1]. The authors emphasize the importance of culturally tailored dietary interventions that align with traditional Asian food habits for improving adherence and effectiveness, as they respect unique dietary preferences and cultural traditions better than Western diets. As a high school Asian advocate for cardiovascular health promotion and disease prevention, I find the implications for future programs that tailor both direct dietary recommendations and educational classes particularly promising.

South Asians experience higher cardiometabolic risks and mortality rates from atherosclerotic cardiovascular disease compared with other Asian subgroups and non-Hispanic White adults [2]. This makes them a key target population for dietary interventions aimed at preventing early plaque buildup in the arteries. However, it is imperative to recognize that the intervention recommendations for Asians should be distinct from those of other ethnic groups, as some families may hesitate to embrace recommended eating habits if they fail to respect cultural food preferences, dietary restrictions, or financial limitations.

The Mediterranean Diet (MD) has been shown to significantly improve cardiometabolic profiles, yet there is a paucity of studies on the MD benefits for non-Mediterranean racial and ethnic minorities, for whom this diet may be unfamiliar, inaccessible, or culturally unacceptable. Just as an MD intervention was culturally tailored to Puerto Ricans by incorporating traditional Puerto Rican foods, such as legumes and vegetable oils [3], similar adaptations can be applied to Asian populations. The traditional Asian diet is rich in fruits, vegetables, and plant-based foods such as soy and lentils, often containing unsweetened tea, fermented foods, and various herbs and spices [1]. Where possible, these culturally relevant options can replace corresponding foods in the original MD, ensuring that interventions align with familiar eating patterns while maintaining heart-healthy benefits.

In addition to simply tailoring the foods themselves, it is crucial to also elucidate the reasoning behind the urgency for adopting these diets through the tailoring of accompanying educational messages. The Kaiser Permanente Northern California Heart Health for South Asians Program is a 2-h educational class offering culturally relevant lifestyle and dietary guidance to South Asian patients, and participation is linked to long-term improvements in cholesterol levels and short-term reductions in blood pressure and body mass index [4]. Educational interventions such as this may consider targeting traditional beliefs and ideas, collectivism, family and kinship ties, fatalism, cultural norms, gender roles, and normative thinking, factors that play fundamental roles in dietary and medication adherence [5].

Specifically for Chinese populations, it may be strategically effective to integrate concepts of Ying–Yang, hot–cold foods related to disease and body balance, traditional Chinese medicine, and cultural idioms and stories [6]. As for the delivery of the interventions, linguistic designs are critical to tailoring to the vast number of dialects across Asia. Furthermore, interventions for physical activity often come hand-in-hand with those for nutrition to support general lifestyle medicine. Continuing with the idea of cultural sensitivity, although soccer may be more appealing for Brazilian communities, exercises such as yoga or Tai Chi may be more suitable and implementable for South Asians [7].

Therefore, merely increasing the funding and supply of health care resources is insufficient to address the disproportionately high burden of cardiovascular disease among South Asians. Instead, health care systems, policymakers, and stakeholders must creatively and deliberately oversee the cultural tailoring of these resources in lifestyle modification to form a nuanced understanding of prevention. Only then can we truly reduce the cardiovascular health care disparities among racial and ethnic minorities, fostering sustainable, long-term improvements in heart health across all populations, regardless of their cultural backgrounds.

## Author contribution

The sole author had responsibility for all parts of the manuscript.

## Conflict of interest

The authors report no conflicts of interest.
